# Dr. Charles S. Meahl

**Published:** 1916-05

**Authors:** 


**Dr. Charles S. Meahl,** Buffalo 1890, died April 17.

